# Latanoprost Induced Iris Pigment Epithelial and Ciliary Body Cyst Formation in Hypermetropic Eyes

**DOI:** 10.1155/2017/9362163

**Published:** 2017-10-08

**Authors:** Abhijit Anand Mohite, Rangarajan V. Prabhu, Thomas Ressiniotis

**Affiliations:** ^1^Department of Ophthalmology, New Cross Hospital, The Royal Wolverhampton Hospitals NHS Trust, Wolverhampton Road, Wolverhampton WV10 0QP, UK; ^2^Department of Ophthalmology, Good Hope Hospital, Heart of England NHS Foundation Trust, Rectory Road, Sutton Coldfield B75 7RR, UK

## Abstract

**Purpose:**

Latanoprost has become one of the most widely prescribed topical antihypertensive medications in recent years. Yet there have been few reports of secondary iris pigment epithelial (IPE) and ciliary body (CB) cyst formation to date and none, to our knowledge, reported in eyes predisposed to primary angle closure.

**Methods:**

We report the first documented case of bilateral IPE and CB cysts in a hypermetropic patient with prior laser peripheral iridotomies (LPIs) as a rare, delayed side effect of topical Latanoprost treatment. The cysts subsided with discontinuation of Latanoprost, thereby demonstrating a causal relationship. We discuss the pathogenesis of such cysts and advocate using of serial ultrasound biomicroscopic (UBM) images to monitor them.

**Results and Conclusions:**

Latanoprost may cause iris pigment epithelial and ciliary body cysts that remain clinically undetected. In eyes predisposed to angle closure, such cysts may become clinically detectable and masquerade as iris tumours. Prior laser peripheral iridotomies in these eyes may delay or prevent the detection of these cysts. Ultrasound biomicroscopy (UBM) imaging is therefore a valuable tool in diagnosing and monitoring these cysts.

## 1. Introduction

Latanoprost is a prostaglandin-F2-alpha analogue that acts as an agonist of the prostaglandin-F2-alpha receptor. In 1996, it became the first prostaglandin analogue available and is currently first-line treatment option for reducing intraocular pressure (IOP) in ocular hypertension or open-angle glaucoma.

Despite its widespread use, there have been no reported cases of secondary ciliary body (CB) cyst formation and only four reported cases of secondary iris pigment epithelial (IPE) cyst formation. In addition, cyst formation is not a listed side effect of the drug in the most recent British National Formulary [1]. We report the first documented case of bilateral IPE and CB cysts in a hypermetrope with prior laser peripheral iridotomies (LPIs) as a delayed side effect of topical Latanoprost.

## 2. Case Report

A 62-year-old Caucasian male was being monitored annually for ocular hypertension, having been referred from his optician in 2006 with raised intraocular pressures on a routine eye test. His subjective refraction was +6.50/−1.25 × 25 in the right eye (OD) and +6.75/−1.00 × 160 in the left eye (OS). Best-corrected visual acuities (BCVAs) were 6/4 and 6/6 in the right and left eyes, respectively. There was no family history of glaucoma, and the patient was asthmatic. Initial gonioscopy had revealed open but narrow, nonoccludable iridotrabecular angles bilaterally.

Presenting IOPs were 24 mmHg and 19 mmHg, with average central corneal thicknesses of 542 (OD) and 555 (OS) microns. Visual fields on the Humphrey 24-2 automated analyser were normal and cup-to-disc ratios were 0.5 each, with healthy neuroretinal rim appearances. In April 2011, the patient was noted to have raised IOPs of 34 mmHg (OD) and 30 mmHg (OS) with gonioscopically narrow, occludable iridotrabecular angles likely as a result of an increase in lens thickness with time. Only the anterior part of the trabecular meshwork was visible in more than half of the angle, with no peripheral anterior synechiae in either eye (all parts of the angle opened fully on indentation gonioscopy in both eyes). His discs and visual fields remained unchanged.

Primary angle closure (PAC) was diagnosed and he underwent routine sequential laser peripheral iridotomies (LPIs). Two months later, IOPs were still raised despite gonioscopic confirmation of opening of the angles. He was therefore started on topical Latanoprost at night to good effect with IOPs lowering to 17 mmHg bilaterally. Visual fields and IOPs remained stable over the next three years, during which period he underwent a number of routine reviews including a dilated fundal examination that was normal.

**Figure 1:**
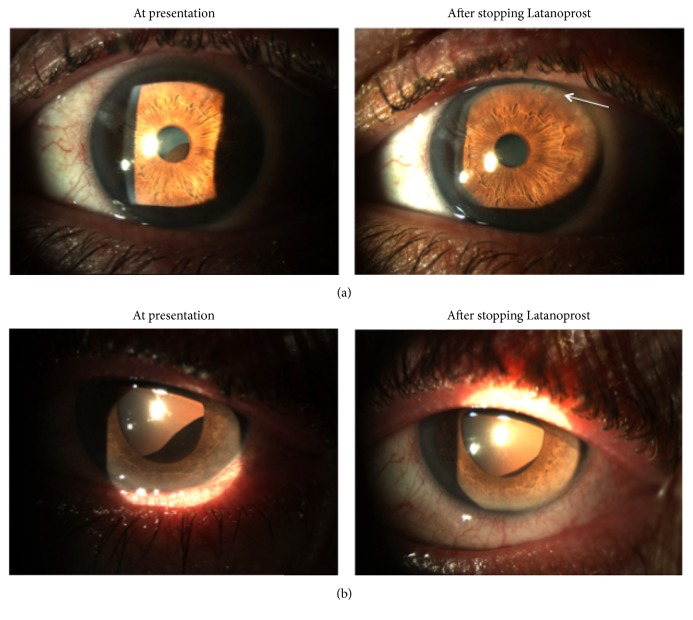
(a) Predilation and (b) postdilation anterior segment photos of left eye showing large inferotemporal iris cyst at presentation, causing ectropion uveae and anterior displacement of the iris surface. Four months after stopping topical Latanoprost, the cyst is only evident after dilation. Note patent peripheral iridotomies (arrow).

At a routine appointment in November 2014, forty-one months after commencing Latanoprost, he was noted to have uveal tissue protruding through the undilated left pupil, causing anterior displacement of the inferotemporal iris surface (Figure 1(a)). The right iris appeared unremarkable. Whilst both eyes were noted to have early lens opacities, BCVAs remained excellent at 6/5 (OD) and 6/7.5 (OS), and IOPs remained stable at 18 mmHg (OD) and 19 mmHg (OS). There were no signs of uveitis and dilated fundal examination was unremarkable in both eyes. The smooth dark brown mass of uveal tissue was more pronounced after pupil dilation (Figure 1(b)).

**Figure 2:**
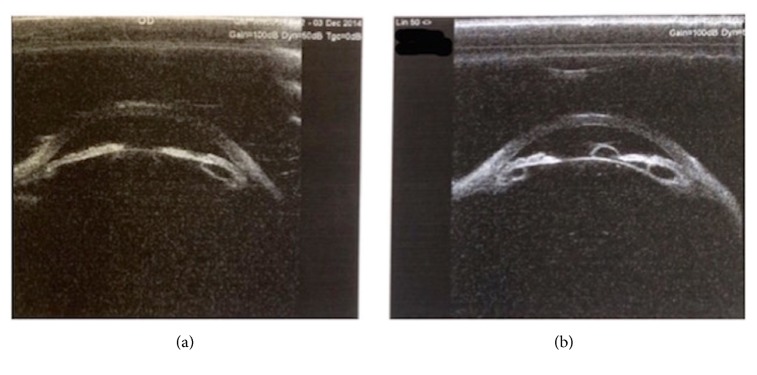
Ultrasound biomicroscopy (UBM) images of (a) right and (b) left eyes showing multiple iris pigment epithelial and ciliary body cysts in both eyes prior to stopping topical Latanoprost.

An ultrasound biomicroscopy (UBM) scan revealed multiple IPE and ciliary body (CB) cysts at the 3, 6, and 9 o'clock meridians in the left eye with the largest being in the inferotemporal quadrant. Similar IPE and CB cysts were detected in the contralateral eye, despite not being evident on the slit lamp (Figure 2). Axial lengths were 20.18 mm (OD) and 19.93 mm (OS), whilst lens thicknesses were 5.17 mm (OD) and 5.05 (OS) mm, resulting in high lens: axial length ratios of 0.25 in each eye.

The patient was referred to an ocular oncologist who felt the appearance was in keeping with benign secondary IPE and CB cysts. Latanoprost was discontinued and Brinzolamide commenced. Beta-blockers were avoided given the patient's asthmatic status. During subsequent monitoring, the left iris cyst was noted to gradually decrease in size and, four months later, was only evident on dilated slit-lamp examination (Figure 1(b)). At the most recent nine-month follow-up, the cyst was not visible even after dilation. IOPs were reasonable at 20 mmHg (OD) and 15 mmHg (OS) on topical Brinzolamide three times daily. Visual fields and optic discs had remained unchanged.

## 3. Discussion

Iris cysts are a rare side effect of topical Latanoprost treatment. It has been suggested that the increase in uveoscleral outflow caused by PG-F2-alpha analogues such as Latanoprost contributes to cyst formation by altering aqueous humour dynamics through the interepithelial space of the posterior iris [2]. This case suggests that high hypermetropia may be an additional mechanism by which these cysts become clinically detectable.

Ours is first reported case of CB cysts occurring together with the IPE cysts. Only two previous cases had UBM imaging to assess the anterior segment's morphological changes caused by the IPE cysts, neither of which were in eyes with PAC and neither of which reported additional CB cyst formation. None of the previous cases documented axial lengths or relative lens sizes.

Four previously reported cases of IPE cysts secondary to Latanoprost have taken between 5 weeks and 18 months to develop [3–6]. Our patient was a high hypermetrope with thick lenses and occludable angles who had LPIs before Latanoprost had been commenced. He developed clinically detectable IPE and CB cysts more than 3 years later, far longer than those previously reported. This delay could be explained if the prior LPIs prevented a relative pupil block and forward bowing of the iris, allowing the cysts to gradually enlarge undetected. In addition, the IOP lowering effect of Latanoprost itself could explain why the cysts were able to enlarge to such an extent over a long period of time, without causing any IOP increase.

We postulate that had our patient not had prior LPIs, the formation of clinically detectable IPE and CB cysts would have been far earlier due to a further increase in sectoral appositional angle closure caused by the developing cysts. This may give rise to a “pseudoplateau iris” syndrome, as has been previously reported [7]. Although a possibility, it is not known whether these sectoral secondary IPE and CB cysts could potentiate angle closure in eyes with narrow angles.

## 4. Conclusions

In the absence of a prospective study utilising serial anterior segment UBM imaging, the true incidence of iris and ciliary body cyst formation due to Latanoprost and other topical prostaglandin analogues is unknown. Ours is the first reported case of bilateral UBM-confirmed secondary ciliary body and IPE cyst formation due to Latanoprost, with the cysts in the fellow eye only being detected by UBM imaging.

We therefore propose that both IPE and CB cysts formations are rare side effects of Latanoprost treatment. In eyes anatomically at risk of PAC, the development of these cysts may potentiate sectoral angle closure and we therefore recommend UBM as the imaging modality of choice to help diagnose and monitor these cysts.
